# Global research dynamics in the Tai Chi and insomnia: a bibliometric study from 2006 to 2025

**DOI:** 10.3389/fpsyt.2025.1669378

**Published:** 2025-11-07

**Authors:** Mo Liao

**Affiliations:** Rehabilitation Center, Dazhou Integrated Traditional Chinese Medicine &Western Medicine Hospital, Dazhou, Sichuan, China

**Keywords:** Tai Chi, insomnia, bibliometric analysis, research trends, complementary and integrative medicine

## Abstract

**Objective:**

Tai Chi has demonstrated beneficial effects in managing insomnia. However, no bibliometric analysis has systematically examined the relationship between Tai Chi and insomnia. This study aims to quantitatively evaluate the global research landscape and emerging trends related to “Tai Chi and insomnia” from 2006 to 2025 using bibliometric methods, thereby offering evidence-based insights and guidance for future basic and clinical research.

**Methods:**

A bibliometric analysis was conducted using data retrieved from the Web of Science Core Collection (WoSCC)) and Scopus databases for the period 2006–2025. The data were processed and visualized using Bibliometrix (R software), VOSviewer, and CiteSpace.

**Results:**

A total of 281 valid articles from the WoSCC and 489 from Scopus were included. The number of publications on Tai Chi and insomnia has steadily increased over the past two decades. China and the United States are the primary contributors to research in this field, forming an international collaboration network centered around the University of California, Los Angeles (UCLA) and the University of Hong Kong. Among them, Professor Michael R. Irwin from UCLA is the most prolific author and maintains the widest collaborative network in this domain. Frontiers in Psychiatry and Sleep Medicine Reviews show the highest publication volumes, whereas Sleep ranks first in citation frequency and functions as a key hub for academic collaboration. Using cluster analysis, keyword frequency assessment, co-word mapping, and thematic evolution analysis, we identified several prominent research hotspots. These hotspots primarily focus on three areas: the clinical application and efficacy evaluation of Tai Chi in specific patient populations with sleep disorders, mechanistic and evidence-based investigations of Tai Chi interventions for insomnia, and comparative or integrative studies of Tai Chi with other non-pharmacological treatments for insomnia.

**Conclusion:**

The therapeutic potential of Tai Chi for insomnia has garnered growing global interest and is poised to become a major focus in insomnia management. This study provides a comprehensive overview of the field’s current status and key research trends, offering valuable direction for future studies.

## 1 Introduction

Insomnia is a complex sleep disorder that poses a major challenge to modern medicine and public health. Global epidemiological data show that around 27%–30% of adults experience insomnia to varying degrees, with 6%–10% meeting the diagnostic criteria for chronic insomnia (1, 2). Insomnia is marked by difficulty falling asleep, poor sleep maintenance, and early awakenings, often accompanied by impaired daytime functioning such as reduced attention, memory decline, and emotional instability, which significantly impair quality of life and productivity (3).

Epidemiological studies reveal substantial variation in insomnia prevalence by age and gender.Among adults aged 65 and older, prevalence rises to 30%–48%, and women are approximately 1.4 times more likely than men to experience insomnia.These disparities are likely due to age-related physiological changes, hormonal fluctuations, and psychosocial influences.Pathophysiological studies have implicated multiple systems in insomnia, including dysregulation of the hypothalamic–pituitary–adrenal (HPA) axis, autonomic nervous system dysfunction, and altered brain connectivity (3).

Insomnia is associated with substantial health consequences. Clinical evidence indicates that individuals with chronic insomnia are 2–3 times more likely to develop depression and 1.5–2 times more likely to experience anxiety disorders. Moreover, insomnia is a significant risk factor for cardiovascular disease and cognitive impairment (4–6).Socioeconomically, the direct medical costs and indirect productivity losses due to insomnia are considerable.According to the American Academy of Sleep Medicine, insomnia results in annual economic losses amounting to hundreds of billions of dollars in the United States alone (7).

Current mainstream treatments for insomnia include pharmacological and psychological-behavioral approaches. Benzodiazepine receptor agonists and related drugs offer short-term efficacy but carry risks of dependence, tolerance, and adverse effects with long-term use (8). CBT-I, the gold-standard non-pharmacological treatment, faces limitations such as limited availability of trained therapists and extended treatment durations.These gaps have fueled growing interest in complementary and alternative therapies like Tai Chi (9, 10).

Tai Chi, a traditional Chinese martial art with a history of more than a century, has recently spread worldwide as a form of physical exercise. It combines slow, flowing movements with controlled deep breathing and focused mental attention to form an integrated system for mind-body regulation (11). Over centuries, various styles of Tai Chi have evolved, and its health benefits are increasingly recognized in modern medicine (12). As a treatment for insomnia, Tai Chi offers distinct advantages: it is low-impact and suitable for all ages; it promotes mind-body integration to balance physical and psychological states; and its adaptable practice settings enhance accessibility (13). A growing body of clinical evidence supports Tai Chi’s efficacy for insomnia. Randomized controlled trials (RCTs) have shown that structured Tai Chi practice improves sleep efficiency, reduces sleep latency, and yields sustained benefits (14, 15). Mechanistically, Tai Chi may improve sleep by enhancing parasympathetic activity, regulating cortisol levels, and improving brain functional connectivity (16).

Bibliometric analysis, a key scientometric tool, enables identification of publication trends, emerging themes, interdisciplinary links, and international collaborations, offering a systematic perspective on research field development (17). This study employed bibliometric methods to systematically analyze publications related to “Tai Chi” and “insomnia” indexed in the Web of Science Core Collection (WoSCC) and Scopus databases from January 1, 2006, to July 14, 2025. Using bibliometric and visualization techniques, we objectively mapped the field’s research output characteristics, collaborative network structures, and the evolution of research hotspots. The selection of these two databases was based on their recognized status as the world’s most authoritative citation resources. Both provide high-quality, well-structured metadata essential for network construction and trend prediction, which are critical for ensuring the reliability and validity of bibliometric analyses. In addition, only English-language publications were included to maintain methodological feasibility. Searches of major Chinese databases, including CNKI and Wanfang, revealed that studies on Tai Chi interventions for insomnia are exceedingly limited, falling far below the threshold required for robust bibliometric analyses such as co-citation mapping or keyword clustering. Conducting such analyses on sparse datasets would likely produce unstable or even misleading results. Therefore, to preserve methodological rigor and ensure the reliability of our findings, Chinese-language literature was excluded. By systematically summarizing research progress in this field, this study not only advances scholarly understanding but also provides scientific evidence to guide clinical practice and inform health policy development.

## 2 Materials and methods

### 2.1 Data collection

Data were collected from the WoSCC and Scopus databases as of July 14, 2025. The WoSCC search strategy was as follows: TS = ((“Tai Chi” OR “Taiji” OR “Tai Ji” OR “Taichi”) AND (insomnia OR “circadian rhythm” OR sleep*)) AND LA = (English) AND DT = (Article OR Review) AND DOP = (2006-01-01/2025-07-14). After removing irrelevant and duplicate records, 281 valid articles were obtained. Data were exported in plain text format, including full records and cited references.

The Scopus search strategy was: TITLE-ABS-KEY (Taichi OR Taiji OR “Tai Ji” OR “Tai chi”) AND TITLE-ABS-KEY (insomnia OR “circadian rhythm” OR sleep*) AND PUBYEAR > 2005 AND PUBYEAR < 2026 AND (LIMIT-TO (DOCTYPE, “ar”) OR LIMIT-TO (DOCTYPE, “re”)) AND (LIMIT-TO (LANGUAGE, “English”)). After filtering, 489 valid articles were included. These records were saved in CSV format with full metadata and cited references.

### 2.2 Data analysis

Bibliometric analysis followed established methodologies (18). Annual publication trends were analyzed using Origin 2018. Further analyses were conducted using R (version 4.5.1) with the Bibliometrix software (version 4.0; http://www.bibliometrix.org) (19), VOSviewer (version 1.6.20) (20), and CiteSpace (version 6.1.4) (21).

In the WoSCC database, the co-authorship network analysis was conducted with a minimum threshold of ≥ 2 publications for countries, ≥ 3 publications for institutions, and ≥ 2 publications for authors. For co-citation analysis, the minimum citation count for source references was ≥ 30.For keyword co-occurrence, the minimum frequency was ≥ 6.

In the Scopus database, the co-authorship network analysis used thresholds of ≥ 2 publications for countries, ≥ 2 publications for institutions, and ≥ 3 publications for authors. For co-citation analysis, the threshold was ≥35 citations. For keyword co-occurrence, the threshold was ≥24, excluding general terms such as “Tai Chi” and “insomnia” along with their synonyms.

Journal impact factors (IF) were retrieved from the 2024 Journal Citation Reports (JCR).

## 3 Results

### 3.1 Overview of research on Tai Chi and insomnia

**Figure 1:**
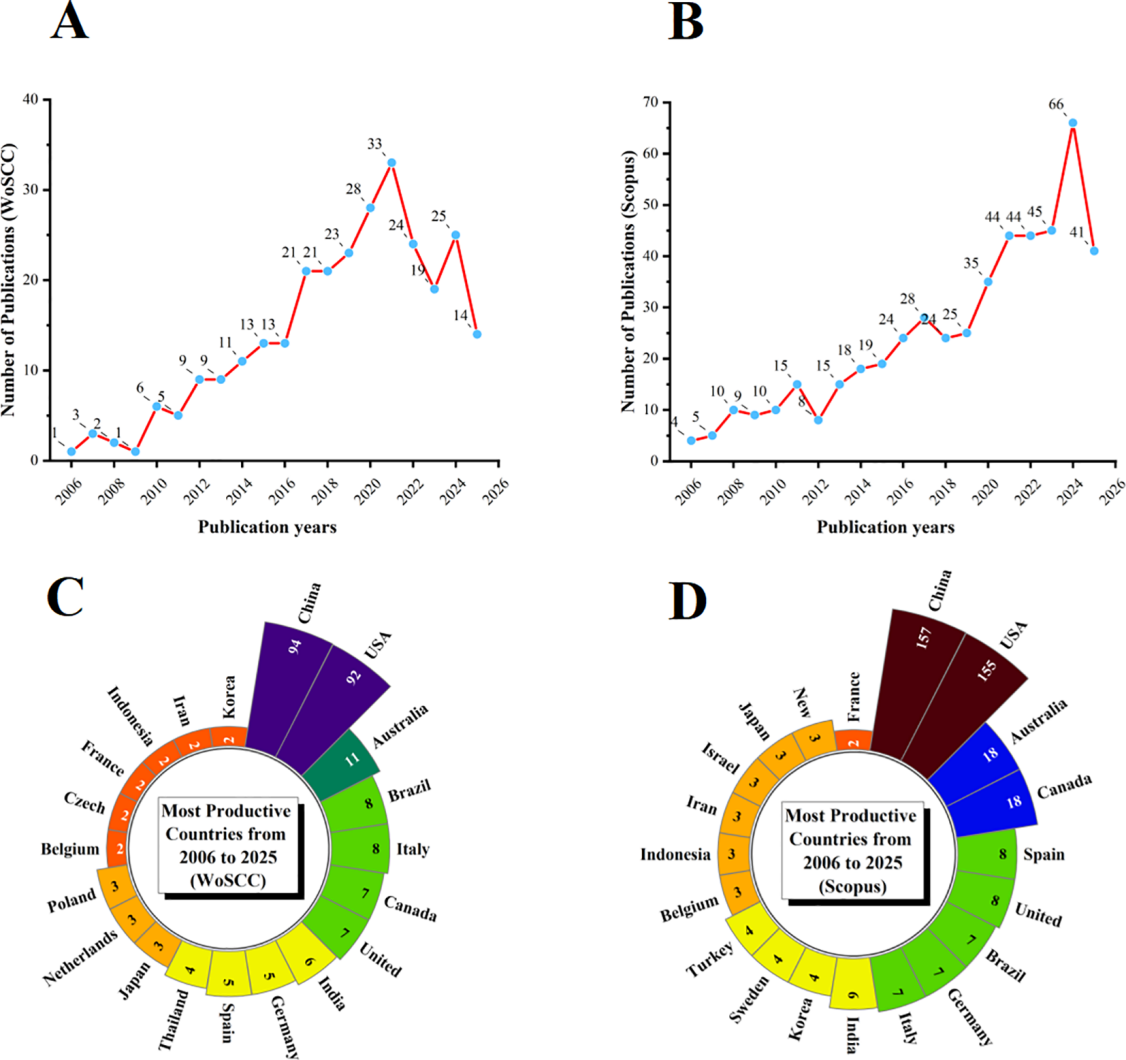
Annual publication trends of Tai Chi and insomnia-related research from 2006 to 2025. **(A)** Annual publication trends in WoSCC. **(B)** Annual publication trends in Scopus. **(C)** Distribution of corresponding authors’ countries in WoSCC. **(D)** Distribution of corresponding authors’ countries in Scopus.

In the WoSCC database, after removing duplicates, 281 unique records were obtained. From 2006 to 2020, the number of publications on Tai Chi and insomnia exhibited a steady upward trend, followed by a slight decline during 2021–2022 and a resurgence in 2024 (**Figure 1A**). In the Scopus database, 489 unique records were retrieved, with publication numbers consistently increasing from 2006 to 2024 (**Figure 1B**). These parallel trends reflect a growing global interest in the relationship between Tai Chi and insomnia.

A country-level analysis revealed that in the WoSCC database, China (n = 94) was the leading contributor, followed by the United States (n = 92), Australia (n = 11), Brazil (n = 8), and Italy (n = 8). Notably, 25.5% of publications from China and 15.2% from the United States involved international collaboration (**Figure 1C**; **Table 1**). In the Scopus database, China (n = 157) and the United States (n = 155) also led in publication volume, followed by Australia (n = 18), Canada (n = 18), and Spain (n = 8). International collaboration accounted for 27.4% of Chinese and 12.9% of American publications (**Figure 1D**; **Table 1**).

**Table 1:** Most relevant countries by corresponding authors.

WoSCC				Scopus			
Country	Articles	MCP	MCP %	Country	Articles	MCP	MCP %
China	94	24	25.5	China	157	43	27.4
USA	92	14	15.2	USA	155	20	12.9
Australia	11	6	54.5	Australia	18	8	44.4
Brazil	8	3	37.5	Canada	18	8	44.4
Italy	8	2	25	Spain	8	1	12.5
Canada	7	2	28.6	United Kingdom	8	1	12.5
United Kingdom	7	1	14.3	Brazil	7	1	14.3
India	6	0	0	Germany	7	2	28.6
Germany	5	2	40	Italy	7	0	0
Spain	5	3	60	India	6	2	33.3
Thailand	4	1	25	Korea	4	2	50
Japan	3	1	33.3	Sweden	4	2	50
Netherlands	3	0	0	Turkey	4	0	0
Poland	3	0	0	Belgium	3	1	33.3
Belgium	2	1	50	Indonesia	3	1	33.3
Czech Republic	2	0	0	Iran	3	0	0
France	2	1	50	Israel	3	1	33.3
Indonesia	2	1	50	Japan	3	1	33.3
Iran	2	0	0	New Zealand	3	1	33.3
Korea	2	2	100	France	2	0	0

MCP, multiple country publications; MCP %, the proportion of multiple country publications to total publications.

**Figure 2:**
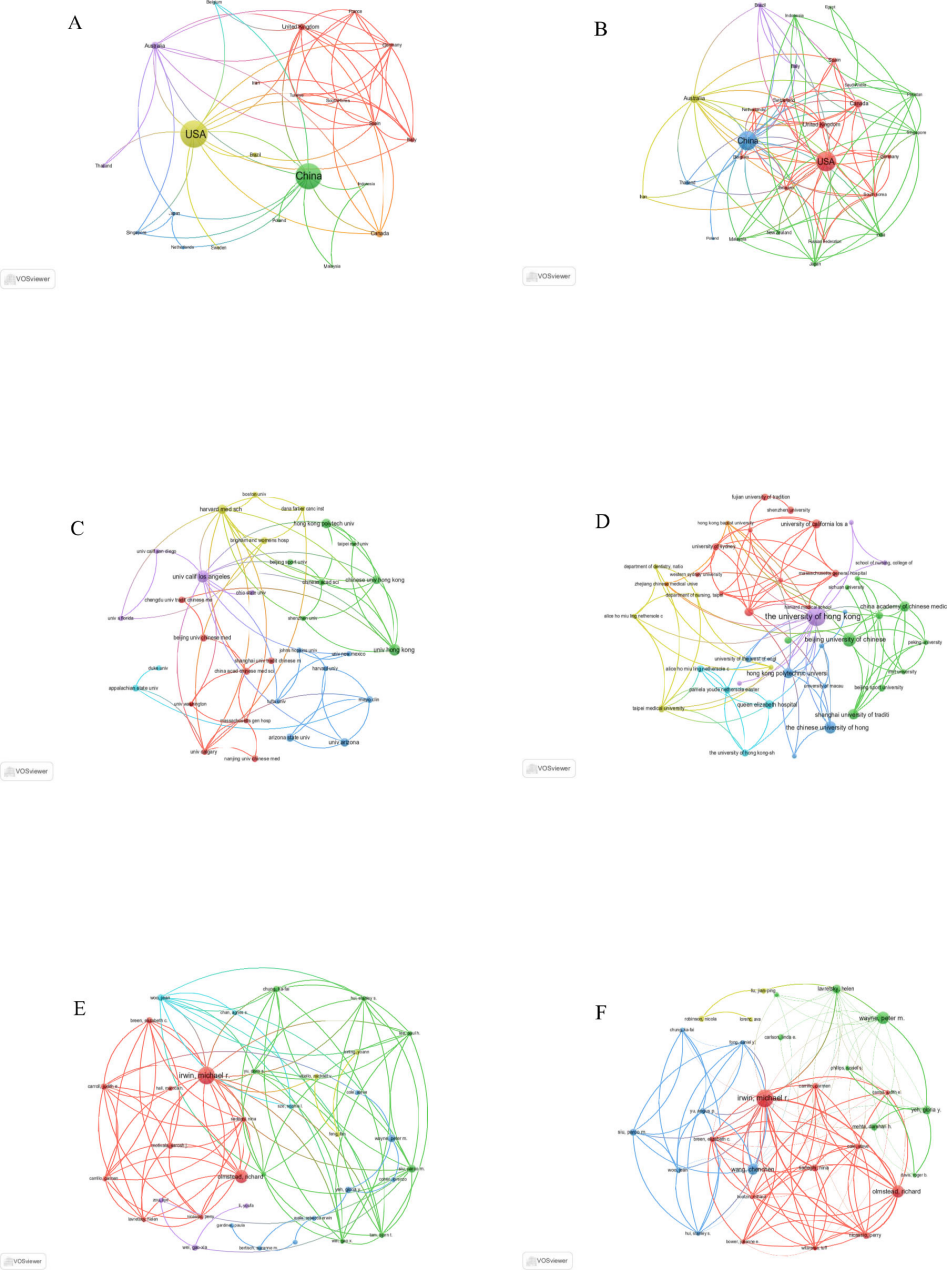
Global bibliometric mapping of research outputs in Tai Chi and insomnia studies (2006–2025): Country distributions, institutional contributions, and author networks. **(A)** Geospatial collaboration network of countries based on WoSCC. **(B)** Geospatial collaboration network of countries based on Scopus. **(C)** Inter-institutional collaboration network derived from WoSCC. **(D)** Inter-institutional collaboration network derived from Scopus. **(E)** Co-authorship network analysis using WoSCC. **(F)** Co-authorship network analysis using Scopus.

Importantly, both China and the United States not only led in publication volume but also maintained extensive global collaboration networks, as illustrated in **Figures 2A, B**. The high research output from these two countries reflects a convergence of cultural, scientific, and societal factors. As the birthplace of Tai Chi, China possesses a strong foundation in traditional medical theory and clinical practice. Governmental support for the modernization of traditional Chinese medicine has further reinforced China’s leading role in this domain. Chinese researchers have predominantly focused on theoretical frameworks and clinical efficacy, particularly emphasizing Tai Chi as a cost-effective solution for the rising incidence of sleep disorders.

By contrast, the United States, leveraging its expertise in neuroscience and sleep medicine, has integrated Tai Chi into the broader framework of complementary and alternative medicine (CAM), emphasizing mechanistic studies and rigorous clinical trials to meet growing demands for non-pharmacological treatments. The complementary strengths of both countries—China contributing traditional knowledge and clinical data, and the U.S. offering advanced research methodologies—have synergistically advanced the field. Future collaboration focused on protocol standardization and mechanistic exploration will be essential for expanding global influence.

In addition, the collaboration maps from both databases indicate that the University of California, Los Angeles (UCLA) and The University of Hong Kong serve as the primary collaborative hubs in the West and East, respectively (**Figures 2C, D**; **Table 2**). These institutions represent the core forces of Western evidence-based medicine and Eastern traditional medicine. Their research philosophies and methodologies are highly complementary and have been continuously supported by funded research projects, thereby fostering sustained, high-level international and cross-cultural collaboration.

**Table 2:** Most relevant affiliations of the relationship between Tai Chi and insomnia.

WoSCC		Scopus	
Affiliation	Articles(*n*)	Affiliation	Articles(*n*)
University of California, Los Angeles	18	The University of Hong Kong	11
The University of Hong Kong	15	Beijing University of Chinese Medicine	10
The Hong Kong Polytechnic University	11	The Chinese University of Hong Kong	6
Harvard Medical School	10	China Academy of Chinese Medical Sciences	5
The Chinese University of Hong Kong	9	Hong Kong Polytechnic University	5
The University of Arizona	9	Shanghai University of Traditional Chinese Medicine	5
Arizona State University	7	Queen Elizabeth Hospital	4
Beijing University of Chinese Medicine	6	Tufts University	4
Appalachian State University	5	University of California Los Angeles	4
Brigham and Women’s Hospital	5	Alice Ho Miu Ling Nethersole Charity Foundation	3
Chengdu University of Traditional Chinese Medicine	5	Beijing Sport University	3
China Academy of Chinese Medical Sciences	5	Fujian University of Traditional Chinese Medicine	3
Chinese Academy of Sciences	5	Lanzhou University	3
Mayo Clinic	5	Massachusetts General Hospital	3
Nanjing University of Chinese Medicine	5	Pamela Youde Nethersole Eastern Hospital	3
Shanghai University of Traditional Chinese Medicine	5	Shenzhen University	3
Beijing Sport University	4	Taipei Medical University	3
University of Calgary	4	The University of Hong Kong-Shenzhen Hospital	3
Boston University	3	University of Sydney	3
Dana-Farber Cancer Institute	3	University of The West Of England	3

Among individual researchers, Michael R. Irwin (University of California, Los Angeles) has long focused on Tai Chi for insomnia and its underlying mechanisms, consistently producing a substantial body of high-quality publications and establishing an extensive international collaboration network. As a result, he stands out as both the most prolific author and the most widely connected collaborator in this field (**Figures 2E, F**; **Table 3**), making him a key influencer in the development of Tai Chi–insomnia research.

**Table 3:** Most relevant authors of the relationship between Tai Chi and insomnia.

WoSCC				
Authors	Articles(n)	Country	Affiliation	Representative papers
Olmstead, Richard	9	USA	University of California, Los Angeles	Cognitive behavioral therapy vs. Tai Chi for late life insomnia and inflammatory risk: a randomized controlled comparative efficacy trial
Irwin, Michael R.	14	USA	University of California, Los Angeles	Cognitive behavioral therapy vs. Tai Chi for late life insomnia and inflammatory risk: a randomized controlled comparative efficacy trial
Siu, Parco M.	4	China	The University of Hong Kong	Effects of Tai Chi or Exercise on Sleep in Older Adults With Insomnia: A Randomized Clinical Trial
Wayne, Peter M.	4	USA	Harvard Medical School	The impact of Tai Chi exercise on balance disorders and falls: a systematic review
Breen, Elizabeth C.	3	USA	University of California, Los Angeles	Prevention of Incident and Recurrent Major Depression in Older Adults With Insomnia: A Randomized Clinical Trial
Carroll, Judith E.	3	USA	University of California, Los Angeles	Sleep Disturbance, Sleep Duration, and Inflammation: A Systematic Review and Meta-Analysis of Cohort Studies and Experimental Sleep Deprivation
Chung, Ka-Fai	3	China	The Chinese University of Hong Kong	Effects of Tai Chi or Exercise on Sleep in Older Adults With Insomnia: A Randomized Clinical Trial
Motivala, Sarosh J.	3	USA	University of California, Los Angeles	Tai chi meditation effects on nuclear factor-κB signaling in lonely older adults: a randomized controlled trial
Wei, Gao-Xia	3	China	Chinese Academy of Sciences	Effects of Mind Body Exercises (Tai Chi/Yoga) on Heart Rate Variability Parameters and Perceived Stress: A Systematic Review with Meta-Analysis of Randomized Controlled Trials
Woo, Jean	3	China	The Chinese University of Hong Kong	Effects of Tai Chi or Exercise on Sleep in Older Adults With Insomnia: A Randomized Clinical Trial
Yeh, Gloria Y.	3	USA	Harvard Medical School	Impact of Exercise Rehabilitation on Exercise Capacity and Quality-of-Life in Heart Failure: Individual Participant Meta-Analysis
Bertisch, Suzanne M.	2	USA	Harvard Medical School	Combination pharmacological therapy targeting multiple mechanisms of sleep apnoea: a randomised controlled cross-over trial
Birling, Yoann	2	Australia	Western Sydney University	Zao Ren An Shen capsule for insomnia: a double-blind, randomized, placebo-controlled trial
Carrillo, Carmen	2	USA	University of California, Los Angeles	Prevention of Incident and Recurrent Major Depression in Older Adults With Insomnia: A Randomized Clinical Trial
Chan, Agnes S.	2	China	The Chinese University of Hong Kong	A Chinese Chan-based mind-body intervention improves psychological well-being and physical health of community-dwelling elderly: a pilot study
Cohen, Lorenzo	2	USA	University of Texas MD Anderson Cancer Center	Qigong/tai chi for sleep and fatigue in prostate cancer patients undergoing radiotherapy: a randomized controlled trial
Cole, Steve	2	USA	University of California, Los Angeles	Short Sleep and Insomnia Are Associated With Accelerated Epigenetic Age
Feng, Fan	2	China	China Academy of Chinese Medical Sciences	The effect of meditative movement on sleep quality: A systematic review
Gardiner, Paula	2	USA	Boston University School of Medicine	Integrative Medicine for Insomnia
Hall, Martica H.	2	USA	University of Pittsburgh School of Medicine	The role of sleep hygiene in promoting public health: A review of empirical evidence
Scopus				
Irwin, Michael R.	13	USA	University of California, Los Angeles	Cognitive behavioral therapy vs. Tai Chi for late life insomnia and inflammatory risk: a randomized controlled comparative efficacy trial
Olmstead, Richard	9	USA	University of California, Los Angeles	Cognitive behavioral therapy vs. Tai Chi for late life insomnia and inflammatory risk: a randomized controlled comparative efficacy trial
Wayne, Peter M.	9	USA	Harvard Medical School	The impact of Tai Chi exercise on balance disorders and falls: a systematic review
Wang, Chenchen	8	USA	Tufts Medical Center	Effect of tai chi versus aerobic exercise for fibromyalgia: comparative effectiveness randomized controlled trial
Lavretsky, Helen	6	USA	University of California, Los Angeles	Cognitive behavioral therapy vs. Tai Chi for late life insomnia and inflammatory risk: a randomized controlled comparative efficacy trial
Yeh, Gloria Y.	6	USA	Harvard Medical School	Impact of Exercise Rehabilitation on Exercise Capacity and Quality-of-Life in Heart Failure: Individual Participant Meta-Analysis
Mehta, Darshan H.	5	USA	Harvard Medical School	Tai Chi and Whole-Person Health
Nicassio, Perry	5	USA	University of California, Los Angeles	Cognitive behavioral therapy vs. Tai Chi for late life insomnia and inflammatory risk: a randomized controlled comparative efficacy trial
Sadeghi, Nina	5	USA	University of California, Los Angeles	Prevention of Incident and Recurrent Major Depression in Older Adults With Insomnia: A Randomized Clinical Trial
Siu, Parco M.	5	China	The University of Hong Kong	Effects of Tai Chi or Exercise on Sleep in Older Adults With Insomnia: A Randomized Clinical Trial
Breen, Elizabeth C.	4	USA	University of California, Los Angeles	Prevention of Incident and Recurrent Major Depression in Older Adults With Insomnia: A Randomized Clinical Trial
Carlson, Linda E.	4	Canada	University of Calgary	Protocol for the MATCH study (Mindfulness and Tai Chi for cancer health): A preference-based multi-site randomized comparative effectiveness trial (CET) of Mindfulness-Based Cancer Recovery (MBCR) vs. Tai Chi/Qigong (TCQ) for cancer survivors
Carrillo, Carmen	4	USA	University of California, Los Angeles	Prevention of Incident and Recurrent Major Depression in Older Adults With Insomnia: A Randomized Clinical Trial
Cole, Steve	4	USA	University of California, Los Angeles	Short Sleep and Insomnia Are Associated With Accelerated Epigenetic Age
Liu, Jian-Ping	4	China	Beijing University of Chinese Medicine	Effects of various exercise interventions in insomnia patients: a systematic review and network meta-analysis
Robinson, Nicola	4	United Kingdom	London South Bank University	Children and Adults Tai Chi Study (CF-CATS2): a randomised controlled feasibility study comparing internet-delivered with face-to-face Tai Chi lessons in cystic fibrosis
Woo, Jean	4	China	The Chinese University of Hong Kong	Effects of Tai Chi or Exercise on Sleep in Older Adults With Insomnia: A Randomized Clinical Trial
Yu, Angus P.	4	China	The University of Hong Kong	Dose-response effects of exercise and caloric restriction on visceral adiposity in overweight and obese adults: a systematic review and meta-analysis of randomised controlled trials
Bootzin, Richard	3	USA	University of Arizona	Cognitive Behavioral Therapy vs. Tai Chi for Late Life Insomnia and Inflammatory Risk: A Randomized Controlled Comparative Efficacy Trial
Bower, Julienne E.	3	USA	University of California, Los Angeles	Tai Chi Chih Compared With Cognitive Behavioral Therapy for the Treatment of Insomnia in Survivors of Breast Cancer: A Randomized, Partially Blinded, Noninferiority Trial

### 3.2 Journal analysis and visualization

To identify the most influential journals in terms of publication output and citation impact in the field of Tai Chi and insomnia, the Bibliometrix package in R was used. Graphical visualizations were generated with the ggplot2 package, while VOSviewer facilitated co-citation analysis.

**Figure 3:**
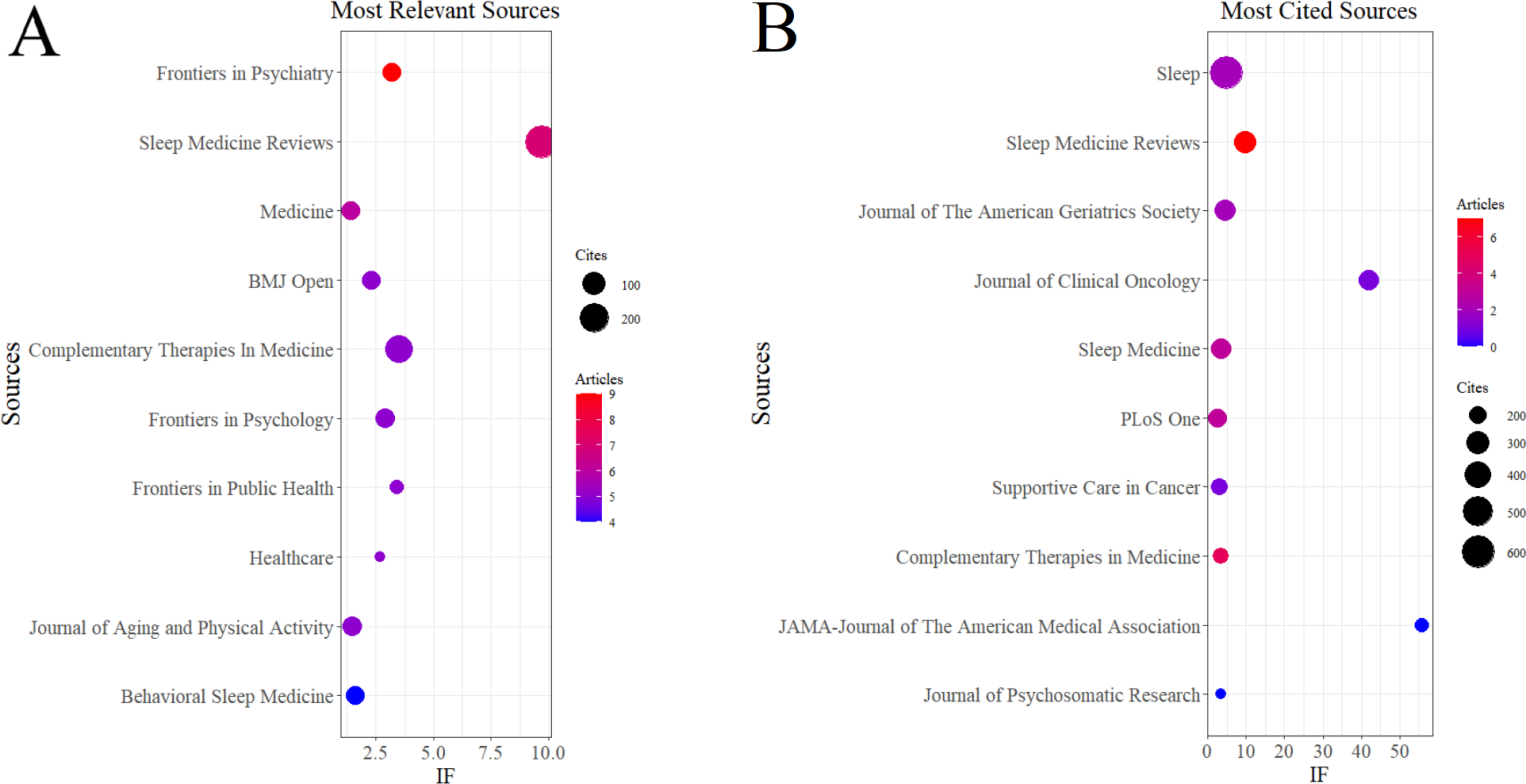
Journal influence analysis in Tai Chi and insomnia research based on WoSCC. **(A)** Journal with the largest number of articles published. **(B)** Journals with the largest number of citations.

In the WoSCC database, our search retrieved a total of 281 publications distributed across 178 academic journals (see **Supplementary Material 1**). As shown in **Table 4** and **Figure 3A**, Frontiers in Psychiatry (n = 9, IF = 3.2) emerged as the leading publication outlet, followed by Sleep Medicine Reviews (n = 7, IF = 9.7), Medicine (n = 6, IF = 1.4), BMJ Open (n = 5, IF = 2.3), and Complementary Therapies in Medicine (n = 5, IF = 3.5). **Table 5** and **Figure 3B** list the most frequently cited journals, with Sleep (n = 603, IF = 4.9) ranking first, followed by Sleep Medicine Reviews (n = 280, IF = 9.7), Journal of the American Geriatrics Society (n = 249, IF = 4.5), Journal of Clinical Oncology (n = 243, IF = 41.9), and Sleep Medicine (n = 243, IF = 3.4).

**Table 4:** Top 10 journals with the most published articles.

WoSCC				Scopus		
Sources	Documents	Cites	IF (2024)	Sources	Documents	IF (2024)
Frontiers in Psychiatry	9	50	3.2	Sleep Medicine Reviews	10	9.7
Sleep Medicine Reviews	7	280	9.7	Complementary Therapies in Medicine	9	3.5
Medicine	6	50	1.4	Integrative Cancer Therapies	9	2.8
BMJ Open	5	46	2.3	Medicine	9	1.4
Complementary Therapies In Medicine	5	177	3.5	BMJ Open	8	2.3
Frontiers in Psychology	5	53	2.9	PLoS One	7	2.6
Frontiers in Public Health	5	16	3.4	Complementary Therapies in Clinical Practice	6	3
Healthcare	5	10	2.7	European Journal of Integrative Medicine	6	1.7
Journal of Aging and Physical Activity	5	56	1.5	Frontiers in Psychiatry	6	3.2
Behavioral Sleep Medicine	4	44	1.6	Journal of Integrative and Complementary Medicine	6	1.7

**Table 5:** Top 10 journals with the most cited journals (WoSCC).

Sources	Cites	Documents	IF (2024)
Sleep	603	2	4.9
Sleep Medicine Reviews	280	7	9.7
Journal of The American Geriatrics Society	249	2	4.5
Journal of Clinical Oncology	243	1	41.9
Sleep Medicine	243	3	3.4
PLoS One	213	3	2.6
Supportive Care in Cancer	186	1	3
Complementary Therapies in Medicine	177	5	3.5
JAMA-Journal of The American Medical Association	158	0	56
Journal of Psychosomatic Research	145	0	3.3

In the Scopus database, a total of 489 publications were identified, spanning 283 academic journals (see **Supplementary Material 2**). As summarized in **Table 4**, Sleep Medicine Reviews (n = 10, IF = 9.7) was the most prominent publishing journal, followed by Complementary Therapies in Medicine (n = 9, IF = 3.5), Integrative Cancer Therapies (n = 9, IF = 2.8), Medicine (n = 9, IF = 1.4), and BMJ Open (n = 8, IF = 2.3).

**Figure 4:**
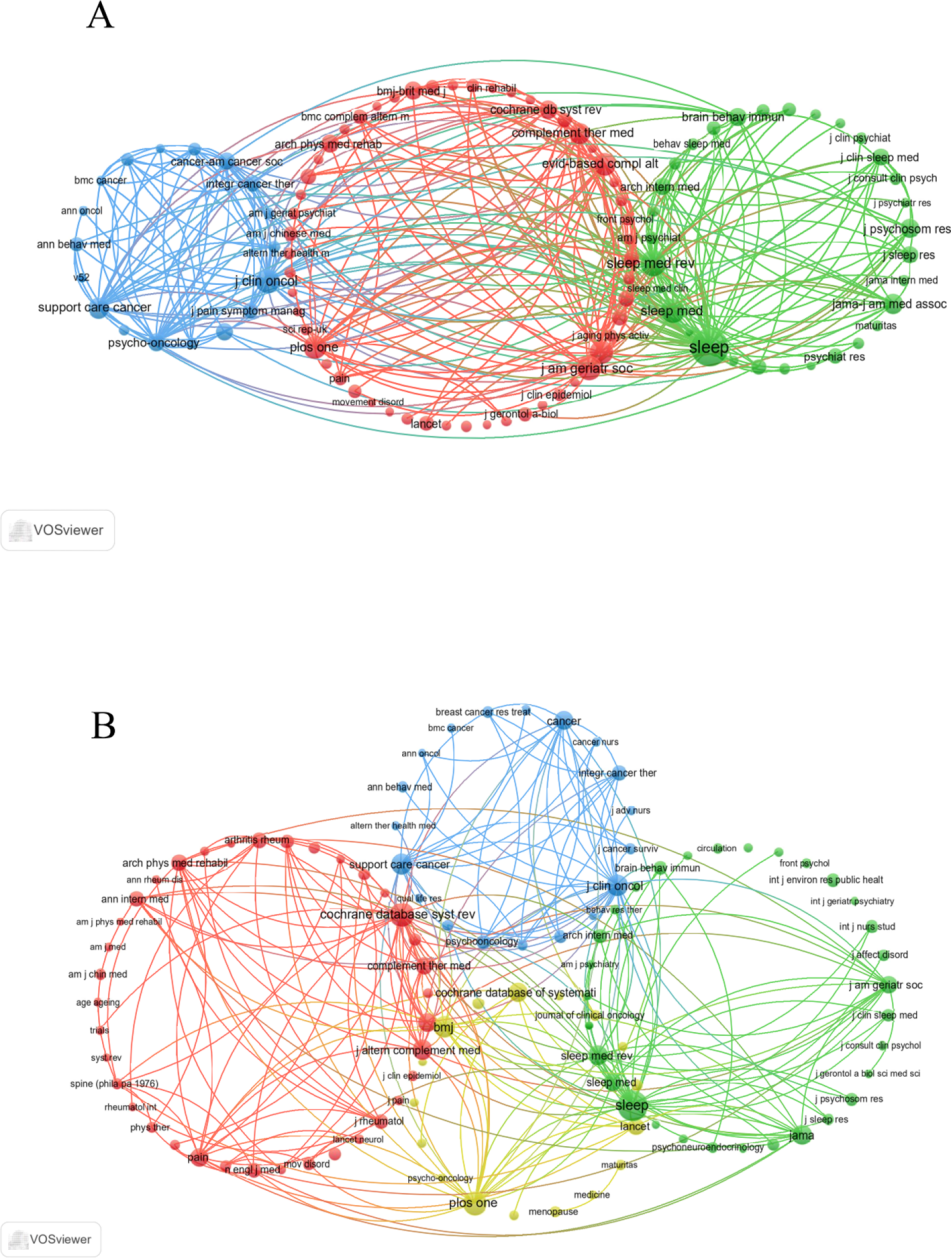
Co-cited journals related to Tai Chi and insomnia. **(A)** Journal co-citation network based on WoSCC. **(B)** Journal co-citation network based on Scopus.

Notably, the co-citation analyses from both WoSCC and Scopus (**Figures 4A, B**) identified Sleep as the central hub of shared references. Collectively, these findings underscore the pivotal roles of Frontiers in Psychiatry, Sleep Medicine Reviews, and Sleep in advancing research on Tai Chi and insomnia.

### 3.3 Most cited references and citation bursts

Using the Bibliometrix package in R, we identified the top 20 most cited articles on Tai Chi and insomnia in the WoSCC database. Each had been cited more than 120 times and were published across 19 different journals (**Table 6**). This dispersion indicates a lack of theoretical consolidation in the field. Notable highly cited articles include: “Exercise interventions on health-related quality of life for cancer survivors,” “Exercise training improves sleep quality in middle-aged and older adults with sleep problems: a systematic review,” and “Implications of sleep disturbance and inflammation for Alzheimer’s disease dementia.”

**Table 6:** Top 20 cited references related to the relationship between Tai Chi and insomnia (WoSCC).

Paper	Title	Total citations
MISHRA SI, 2012, COCHRANE DB SYST REV	Exercise interventions on health-related quality of life for cancer survivors	507
YANG PY, 2012, J PHYSIOTHER	Exercise training improves sleep quality in middle-aged and older adults with sleep problems: a systematic review	421
IRWIN MR, 2019, LANCET NEUROL	Implications of sleep disturbance and inflammation for Alzheimer’s disease dementia	370
AMBROSE KR, 2015, BEST PRACT RES CL RH	Physical exercise as non-pharmacological treatment of chronic pain: Why and when	318
BLACK DS, 2015, JAMA INTERN MED	Mindfulness meditation and improvement in sleep quality and daytime impairment among older adults with sleep disturbances: a randomized clinical trial	299
IRWIN MR, 2014, SLEEP	Cognitive behavioral therapy vs. Tai Chi for late life insomnia and inflammatory risk: a randomized controlled comparative efficacy trial	213
SARRIS J, 2011, SLEEP MED REV	A systematic review of insomnia and complementary medicine	210
IRWIN MR, 2008, SLEEP	Improving sleep quality in older adults with moderate sleep complaints: A randomized controlled trial of Tai Chi Chih	192
CALDWELL K, 2010, J AM COLL HEALTH	Developing mindfulness in college students through movement-based courses: effects on self-regulatory self-efficacy, mood, stress, and sleep quality	188
HARTESCU I, 2015, J SLEEP RES	Increased physical activity improves sleep and mood outcomes in inactive people with insomnia: a randomized controlled trial	171
TAYLOR-PILIAE RE, 2014, ARCH PHYS MED REHAB	Effect of Tai Chi on physical function, fall rates and quality of life among older stroke survivors	158
WAYNE PM, 2018, J CANCER SURVIV	Tai Chi and Qigong for cancer-related symptoms and quality of life: a systematic review and meta-analysis	156
IRWIN MR, 2017, J CLIN ONCOL	Tai Chi Chih Compared With Cognitive Behavioral Therapy for the Treatment of Insomnia in Survivors of Breast Cancer: A Randomized, Partially Blinded, Noninferiority Trial	146
NGUYEN MH, 2012, CLIN INTERV AGING	A randomized controlled trial of Tai chi for balance, sleep quality and cognitive performance in elderly Vietnamese	141
BANNO M, 2018, PEERJ	Exercise can improve sleep quality: a systematic review and meta-analysis	141
VANDERLINDEN J, 2020, INT J BEHAV NUTR PHY	Effects of physical activity programs on sleep outcomes in older adults: a systematic review	136
WANG CC, 2018, BMJ-BRIT MED J	Effect of tai chi versus aerobic exercise for fibromyalgia: comparative effectiveness randomized controlled trial	135
AVANCINI A, 2020, ONCOLOGIST	Physical Activity and Exercise in Lung Cancer Care: Will Promises Be Fulfilled?	124
SUNGKARAT S, 2018, NEUROREHAB NEURAL RE	Tai Chi Improves Cognition and Plasma BDNF in Older Adults With Mild Cognitive Impairment: A Randomized Controlled Trial	121
MÖLLER UO, 2019, BMC CANCER	A comprehensive approach to rehabilitation interventions following breast cancer treatment - a systematic review of systematic reviews	121

From the Scopus database, the top 20 most cited articles each had over 150 citations and were published across 18 journals (**Table 7**). Key publications included: “Noninvasive Treatments for Acute, Subacute, and Chronic Low Back Pain: A Clinical Practice Guideline from the American College of Physicians,” “Chronic pain: an update on burden, best practices, and new advances,” and “Psychoneuroimmunology meets neuropsychopharmacology: translational implications of the impact of inflammation on behavior.” These articles offer comprehensive insights into the link between Tai Chi and insomnia.

**Table 7:** Top 20 cited references related to the relationship between Tai Chi and insomnia (Scopus).

Paper	Title	Total citations
QASEEM A, 2017, ANN INTERN MED	Noninvasive Treatments for Acute, Subacute, and Chronic Low Back Pain: A Clinical Practice Guideline From the American College of Physicians	2187
COHEN SP, 2021, LANCET	Chronic pain: an update on burden, best practices, and new advances	1436
HAROON E, 2012, NEUROPSYCHOPHARMACOLOGY	Psychoneuroimmunology meets neuropsychopharmacology: translational implications of the impact of inflammation on behavior	797
SCOTT AJ, 2021, SLEEP MED REV	Improving sleep quality leads to better mental health: A meta-analysis of randomised controlled trials	550
SARZI-PUTTINI P, 2020, NAT REV RHEUMATOL	Fibromyalgia: an update on clinical characteristics, aetiopathogenesis and treatment	481
MISHRA SI, 2012, COCHRANE DATABASE SYST REV	Exercise interventions on health-related quality of life for cancer survivors	406
ASMUNDSON GJG, 2013, DEPRESSION ANXIETY	Let’s get physical: a contemporary review of the anxiolytic effects of exercise for anxiety and its disorders	275
SARRIS J, 2011, SLEEP MED REV	A systematic review of insomnia and complementary medicine	261
BUSCH AJ, 2011, CURR PAIN HEADACHE REP	Exercise therapy for fibromyalgia	256
MAKRIS UE, 2014, JAMA	Management of persistent pain in the older patient: a clinical review	254
IRWIN MR, 2014, SLEEP	Cognitive behavioral therapy vs. Tai Chi for late life insomnia and inflammatory risk: a randomized controlled comparative efficacy trial	237
CALDWELL K, 2010, J AM COLL HEALTH	Developing mindfulness in college students through movement-based courses: effects on self-regulatory self-efficacy, mood, stress, and sleep quality	220
IRWIN MR, 2008, SLEEP	Improving sleep quality in older adults with moderate sleep complaints: A randomized controlled trial of Tai Chi Chih	211
DENG GE, 2009, J SOC INTEGR ONCOL	Evidence-based clinical practice guidelines for integrative oncology: Complementary therapies and botanicals	206
WAYNE PM, 2018, J CANCER SURVIVORSHIP	Tai Chi and Qigong for cancer-related symptoms and quality of life: a systematic review and meta-analysis	182
YUAN Q-L, 2015, PLOS ONE	Traditional Chinese medicine for neck pain and low back pain: a systematic review and meta-analysis	180
IRWIN MR, 2015, BIOL PSYCHIATRY	Cognitive behavioral therapy and tai chi reverse cellular and genomic markers of inflammation in late-life insomnia: a randomized controlled trial	178
WANG C, 2018, BMJ (ONLINE)	Effect of tai chi versus aerobic exercise for fibromyalgia: comparative effectiveness randomized controlled trial	154
TAO W-W, 2016, J PAIN SYMPTOM MANAGE	Effects of Acupuncture, Tuina, Tai Chi, Qigong, and Traditional Chinese Medicine Five-Element Music Therapy on Symptom Management and Quality of Life for Cancer Patients: A Meta-Analysis	153
NGUYEN MH, 2012, CLIN INTERVENTIONS AGING	A randomized controlled trial of Tai chi for balance, sleep quality and cognitive performance in elderly Vietnamese	153

These highly cited papers exert substantial influence in Tai Chi–insomnia research because their themes closely align with key issues such as exercise-based interventions, sleep disorders, and inflammatory mechanisms, while also addressing global public health priorities including cancer rehabilitation, chronic pain, and Alzheimer’s disease. Their readership spans multiple disciplines, including neuroscience, psychology, pain medicine, and rehabilitation. Most are systematic reviews, clinical guidelines, or high-quality clinical trials with strong evidence grades, providing authoritative support for the role of exercise interventions in improving sleep and related conditions. Furthermore, their theoretical discussions on the interplay between inflammation and sleep, as well as immune–neuropsychological interactions, offer a robust scientific basis for understanding how Tai Chi may alleviate insomnia by modulating immune and inflammatory pathways. Collectively, these factors have driven their broad and enduring academic impact in this field.

**Figure 5:**
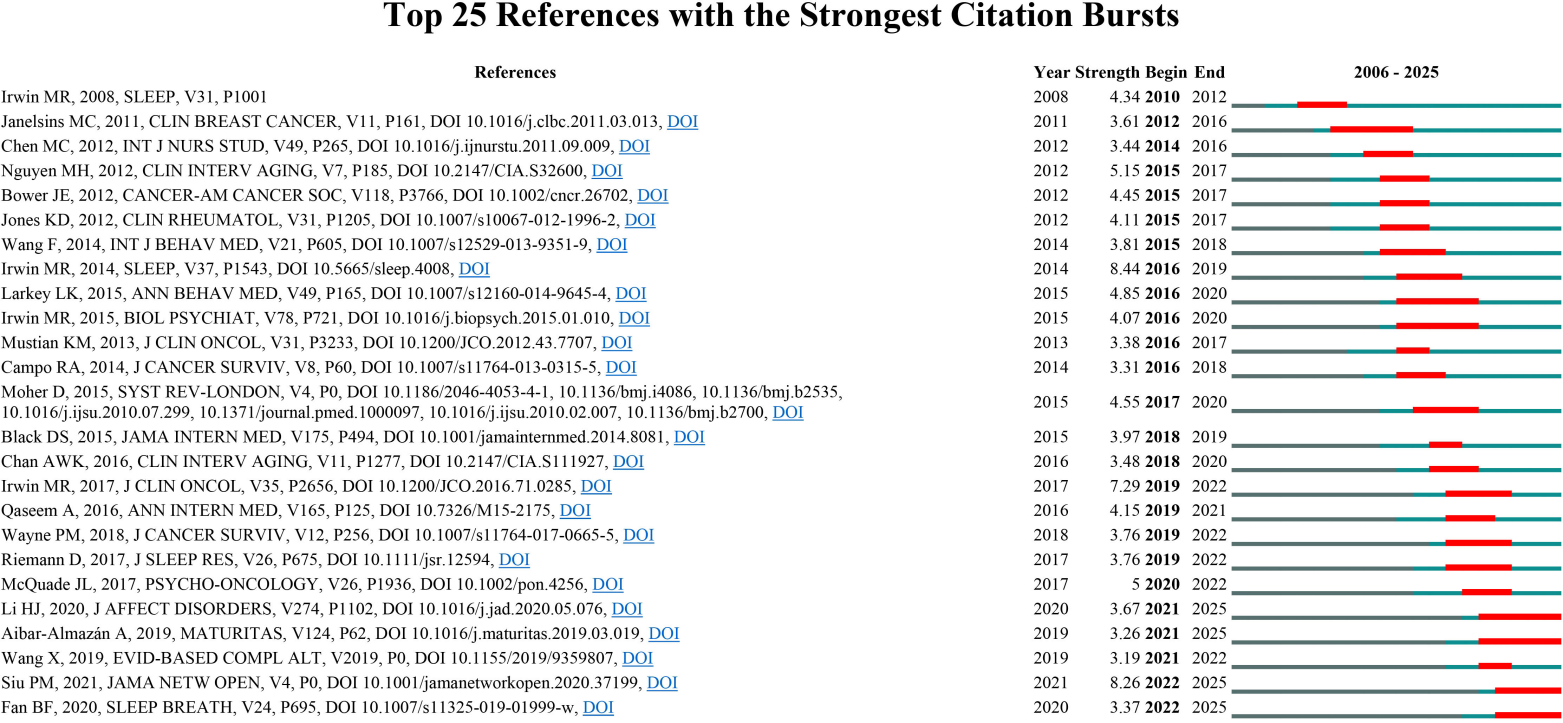
Top 25 references with the strongest citation bursts in Tai Chi and insomnia based on WoSCC.

To further identify research frontiers and emerging trends, we employed CiteSpace to detect the top 25 references with the strongest citation bursts in the WoSCC database (**Figure 5**). The titles and DOIs of these burst references are provided in **Supplementary Material 3**. The three strongest citation bursts were (1): “Cognitive behavioral therapy vs. Tai Chi for late life insomnia and inflammatory risk: a randomized controlled comparative efficacy trial” (Strength = 8.44) (2); “Effects of Tai Chi or exercise on sleep in older adults with insomnia: a randomized clinical trial” (Strength = 8.26); and (3) “Tai Chi Chih compared with cognitive behavioral therapy for the treatment of insomnia in survivors of breast cancer: a randomized, partially blinded, noninferiority trial” (Strength = 7.29). The three most recent bursts were associated with (1): “The efficacy of mind-body (Baduanjin) exercise on self-reported sleep quality and quality of life in elderly subjects with sleep disturbances: a randomized controlled trial” (2); “Effects of Tai Chi or exercise on sleep in older adults with insomnia: a randomized clinical trial”; and (3) “The effect of mind-body therapies on insomnia: a systematic review and meta-analysis.”

These citation-burst papers have attracted substantial attention in a short period largely because of the novelty and timeliness of their study designs, interventions, and clinical implications. First, all employed RCTs or systematic reviews/meta-analyses, providing a high level of evidence. They directly evaluated the efficacy of Tai Chi and other mind-body exercises for insomnia and, in several cases, conducted head-to-head comparisons with established first-line treatments such as cognitive behavioral therapy, thereby offering clinically actionable alternatives or adjunctive options. Second, these investigations targeted high-risk populations—including older adults and cancer survivors—who have a high prevalence of insomnia and limited pharmacologic treatment options, lending their findings considerable public health and rehabilitation relevance. Third, beyond demonstrating improvements in sleep quality, these studies explored underlying mechanisms such as inflammation and immune regulation, providing a contemporary biomedical framework to explain the therapeutic effects of Tai Chi and related traditional mind-body practices. By integrating high-level evidence, clear clinical applicability, and mechanistic insights, these papers rapidly achieved sustained scholarly attention and citation, advancing the internationalization and standardization of Tai Chi as a treatment for insomnia.

### 3.4 Keyword clusters, keyword bursts, and thematic evolution

**Figure 6:**
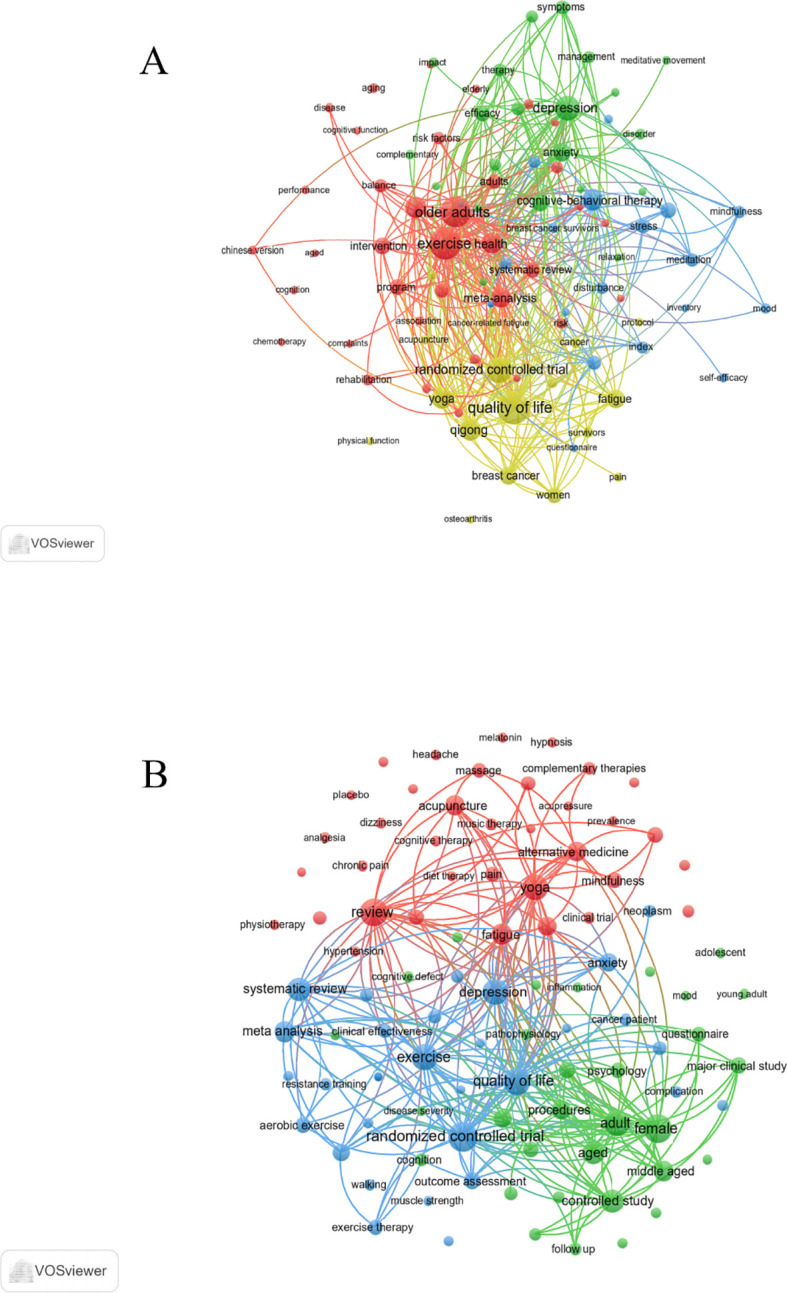
Keyword co-occurrence map of publications in Tai Chi and insomnia. **(A)** Keyword co-occurrence analysis based on WoSCC. **(B)** Keyword co-occurrence analysis based on Scopus.

Keyword clustering provides a concise overview of core themes and research directions within a field. In this study, 1,211 keywords were identified from the WoSCC database using VOSviewer. **Table 8** presents the top 20 most frequently occurring keywords (frequency > 23), revealing major areas of focus. Among them, Quality of Life (n = 100) was the most frequent, followed by Exercise (n = 94), Older Adults (n = 85), Randomized Controlled Trial (n = 66), and Depression (n = 58). Cluster analysis identified four distinct keyword clusters, visualized in **Figure 6A** (1): The red cluster (36 keywords) includes exercise, older adults, health, physical activity, meta-analysis, and intervention, centering on Tai Chi’s role in promoting physical health—particularly cardiovascular benefits—in older adults, aiming to reduce age-related health risks and enhance overall wellness (2). The green cluster (21 keywords) encompasses depression, anxiety, quality, efficacy, symptoms, and mental health, highlighting research on Tai Chi’s efficacy in alleviating psychological disorders, with a focus on its function as a mind-body therapeutic approach (3). The blue cluster (19 keywords) includes cognitive-behavioral therapy, stress reduction, meditation, mindfulness, disturbance, and inflammation, representing mindfulness-based interventions that address emotional regulation, stress, and inflammatory pathways associated with sleep quality improvement (4). The yellow cluster (18 keywords) comprises quality of life, randomized controlled trial, qigong, yoga, fatigue, and breast cancer, emphasizing evidence-based evaluations of Tai Chi and related therapies in improving quality of life, alleviating fatigue, and supporting cancer rehabilitation—especially in addressing insomnia linked to pain and functional impairment. All keywords are detailed in **Supplementary Material 4**.

**Table 8:** Top 20 keywords related to the relationship between Tai Chi and insomnia.

WoSCC		Scopus	
Words	Occurrences	Words	Occurrences
Quality of Life	100	Randomized Controlled Trial	226
Exercise	94	Female	207
Older Adults	85	Review	203
Randomized Controlled Trial	66	Quality of Life	194
Depression	58	Exercise	186
Qigong	46	Adult	171
Health	43	Yoga	171
Cognitive-Behavioral Therapy	42	Depression	170
Physical Activity	41	Systematic Review	146
Yoga	41	Controlled Study	138
Meta-Analysis	40	Aged	130
Anxiety	33	Meta Analysis	117
Fatigue	31	Fatigue	114
Breast Cancer	29	Middle Aged	113
Quality	29	Alternative Medicine	105
Intervention	28	Acupuncture	100
Efficacy	23	Priority Journal	98
Program	23	Procedures	98
Stress Reduction	23	Physical Activity	92
Women	23	Anxiety	89

From the Scopus database, 4,685 keywords were extracted using VOSviewer. **Table 8** lists the top 20 most frequent terms (frequency > 89), with Randomized Controlled Trial (n = 226) as the most common, followed by Female (n = 207), Review (n = 203), Quality of Life (n = 194), and Exercise (n = 186). Cluster analysis revealed three major groups (**Figure 6B**) (1): The red cluster (36 keywords) includes review, yoga, fatigue, alternative medicine, acupuncture, mindfulness, meditation, cognitive behavioral therapy, pain, and mind-body therapies, underscoring the prominence of CAM strategies aimed at alleviating symptoms—such as fatigue, pain, and stress—that exacerbate insomnia (2). The green cluster (31 keywords) includes female, adult, controlled study, aged, middle aged, physical activity, treatment outcome, mental health, questionnaire, and cognition, reflecting clinical studies focused on adult and elderly populations, examining the influence of psychological and cognitive factors, as well as physical activity, on insomnia-related mental health outcomes (3). The blue cluster (30 keywords) includes randomized controlled trial, quality of life, exercise, depression, systematic review, meta-analysis, anxiety, kinesiotherapy, breast cancer, and resistance training, emphasizing evidence-based assessments of exercise therapies—such as Tai Chi—in improving sleep, mood, and life quality, particularly for patients with chronic diseases like cancer. All keyword data are provided in **Supplementary Material 5**.

**Figure 7:**
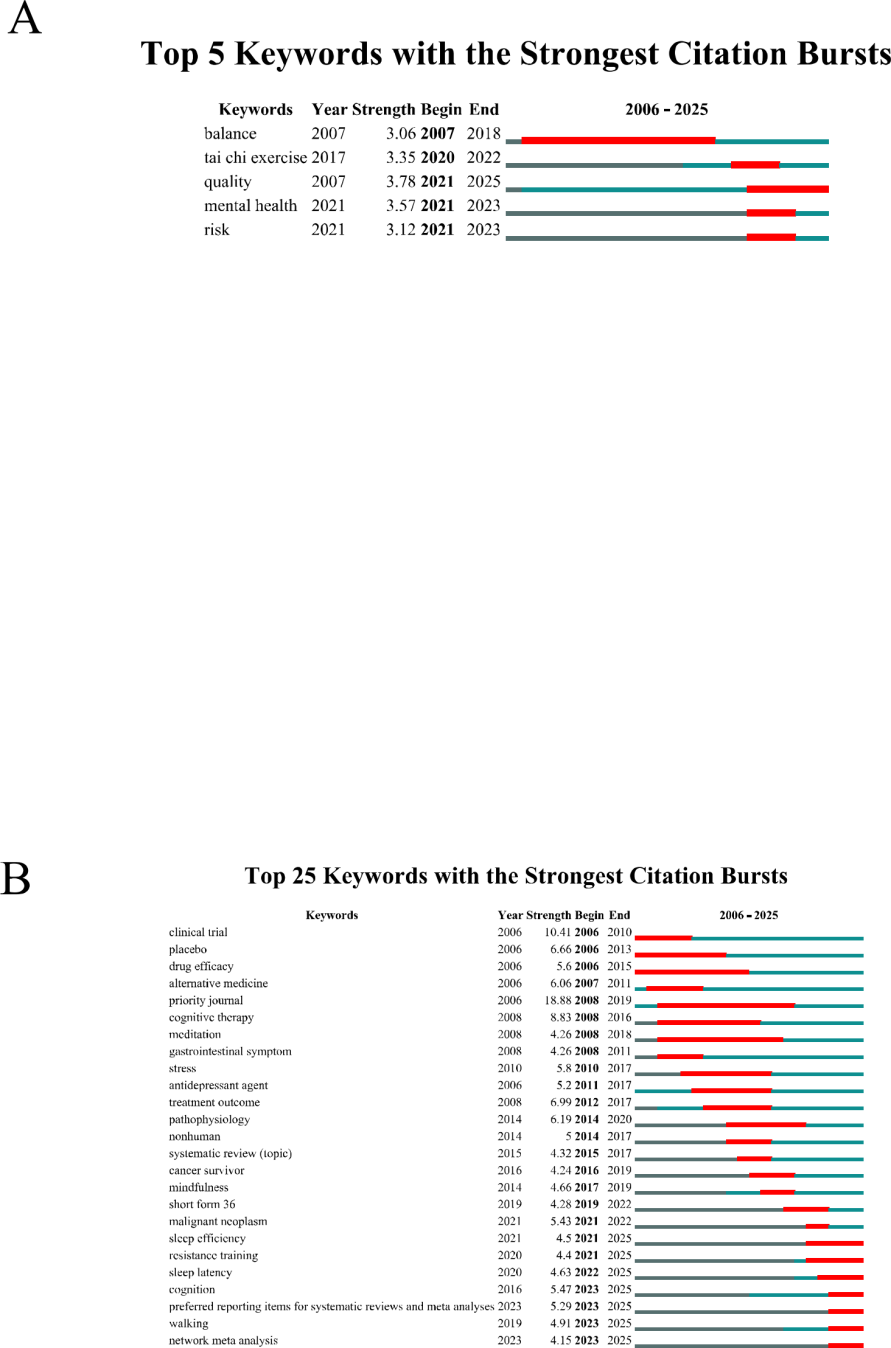
Keyword Bursts of Tai Chi Intervention for Insomnia. **(A)** Keyword Bursts in WoSCC. **(B)** Keyword Bursts in Scopus.

To further explore emerging frontiers and research foci in Tai Chi and insomnia, we used CiteSpace to identify the top five keywords with the strongest citation bursts in the WoSCC database (**Figure 7A**). Notably, the three most prominent burst keywords were quality (strength = 3.78), mental health (strength = 3.57), and tai chi exercise (strength = 3.35), indicating that research attention has expanded from sleep improvement alone to broader health outcomes. The keyword quality underscores the importance of both sleep quality and overall quality of life, highlighting the shift toward patient-centered, comprehensive outcomes. Mental health reflects the close association between insomnia and psychological conditions such as anxiety and depression; with its combined exercise and meditative components, Tai Chi can regulate emotions and improve sleep. Tai chi exercise captures the global trend toward standardizing Tai Chi as a structured exercise intervention. In addition, the three most recent frontier burst keywords—risk, mental health, and quality—further reveal a growing focus on insomnia risk factors, psychological health interventions, and quality-of-life enhancement. These findings suggest that Tai Chi is increasingly viewed as a safe, holistic, non-pharmacological treatment, with research shifting from efficacy validation toward risk management and health promotion.

Similarly, CiteSpace identified the top 25 keyword bursts related to Tai Chi and insomnia in the Scopus database (**Figure 7B**). The three strongest citation bursts were priority journal (strength = 18.88), clinical trial (strength = 10.41), and cognitive therapy (strength = 8.83). The most recent frontier bursts included network meta-analysis, walking, and preferred reporting items for systematic reviews and meta-analyses (PRISMA). These patterns indicate that research on Tai Chi and insomnia is progressively moving toward standardization, clinical application, and methodological rigor. Investigators are focusing not only on treatment outcomes but also on strengthening scientific approaches through clinical trials, network meta-analyses, and adherence to PRISMA guidelines, thereby providing a stronger evidence base for the clinical application of Tai Chi in insomnia management.

**Figure 8:**
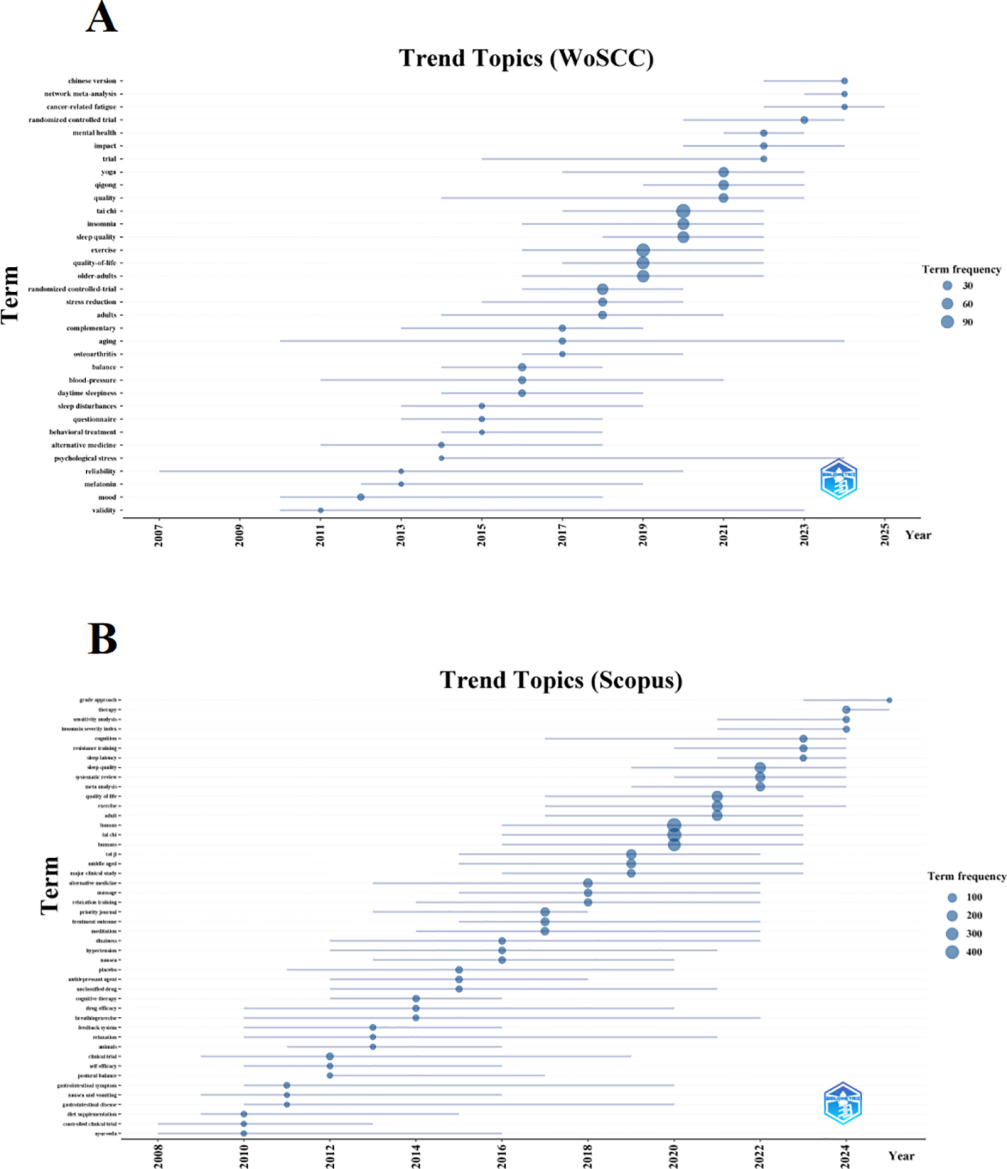
Trend topics on Tai Chi and insomnia. **(A)** Trend topics based on WoSCC. **(B)** Trend topics based on Scopus.

To forecast future research trends, we constructed a dynamic thematic evolution map using the bibliometrix package in the R environment. Data from the WoSCC database are presented in **Figure 8A** and **Supplementary Material 6**. The thematic progression of Tai Chi–insomnia research in WoSCC shows a continuous transition from basic studies to clinical trials and, more recently, to integrative expansion. During the early stage (2006–2014), research focused on the physiological and psychological mechanisms of sleep disorders and the validation of assessment tools, as reflected by keywords such as validity, reliability, questionnaire, and sleep disturbances. Terms like mood, melatonin, and blood pressure indicate early efforts to explain Tai Chi’s effects through emotional regulation and physiological markers. In the middle stage (2015–2019), the emergence of terms such as randomized controlled trial, trial, stress reduction, balance, and adults signaled a shift toward clinical validation centered on randomized trials. Target populations expanded from healthy adults to individuals with sleep disorders or psychological stress, with growing attention to quality-of-life outcomes. In the most recent stage (2020–2025), research diversified further. Core terms such as tai chi, exercise, insomnia, and sleep quality established Tai Chi as a principal intervention, while the appearance of qigong, yoga, mental health, cancer-related fatigue, and network meta-analysis reflects integration with other mind-body therapies, applications to elderly and cancer populations, and the adoption of advanced evidence-synthesis methods to strengthen the evidence base.

Data from the Scopus database (**Figure 8B**, **Supplementary Material 7**) reveal a similar but distinct trajectory. In the early stage (2006–2014), research centered on foundational exploration and methodological validation, with high-frequency terms such as controlled clinical trial, diet supplementation, self-efficacy, breathing exercise, and cognitive therapy, reflecting feasibility testing and preliminary mechanistic investigations. During the middle stage (2015–2019), studies increased in scale and rigor, featuring terms such as Tai Chi, major clinical study, treatment outcome, massage, and relaxation training. This phase emphasized randomized trials to confirm Tai Chi’s benefits for sleep and broader health indicators such as balance and blood pressure, consolidating its role as a key complementary intervention. In the most recent stage (2020–2025), research entered a phase of diversification and high-level evidence synthesis. Emerging terms such as systematic review, meta-analysis, sensitivity analysis, GRADE approach, sleep quality, sleep latency, and insomnia severity index highlight the widespread adoption of advanced evidence-based methods and more refined outcome assessments. Intervention strategies have also expanded to include combinations with exercise, resistance training, and other therapies. Overall, the field has progressed from preliminary feasibility testing to multidimensional, high-quality evidence evaluation, underscoring the growing clinical value of Tai Chi in the prevention and management of insomnia.

### 3.5 Integrated hotspot analysis

In summary, comprehensive analyses of citation bursts, keyword frequency, clustering, and thematic evolution revealed the major research hotspots in the Tai Chi and insomnia domain. Our analysis revealed that research in this field primarily converges on three key thematic directions (1): the clinical application of Tai Chi and the verification of its therapeutic efficacy for sleep disorders in specific populations (2); mechanistic and evidence-based investigations exploring how Tai Chi interventions alleviate insomnia; and (3) comparative and integrative studies examining Tai Chi alongside other non-pharmacological therapies for insomnia management.

## 4 Discussion

This study presents a comprehensive bibliometric analysis of publications on Tai Chi interventions for insomnia indexed in the WoSCC and Scopus from 2006 to 2025. By examining publication trends, contributing countries, journal distribution, highly cited articles, and keyword evolution, we mapped the global landscape, identified key regions of activity, and outlined future research directions in this field.

### 4.1 General information

A total of 281 WoSCC and 489 Scopus articles were included, spanning 2006–2025. The analysis revealed a steady increase in publications on Tai Chi and insomnia, reflecting growing scholarly interest in Tai Chi as a strategy for sleep health. This upward trend not only demonstrates sustained research attention but also underscores the potential of Tai Chi as a widely recognized non-pharmacological intervention for insomnia.

China and the United States emerged as the leading contributors, with publication outputs far surpassing those of other countries. This dominance reflects distinct cultural, scientific, and policy advantages. As the birthplace of Tai Chi, China benefits from a strong foundation in traditional medicine and extensive clinical practice, supported by government policies promoting the modernization of Traditional Chinese Medicine and the dissemination of wellness culture. Consequently, Chinese scholars have made significant contributions to theoretical development and clinical validation, emphasizing Tai Chi’s holistic, accessible, and cost-effective role in addressing the rising prevalence of sleep disorders.

In contrast, U.S. research has drawn on strengths in neuroscience, sleep medicine, and evidence-based methodologies, positioning Tai Chi within the broader framework of complementary and integrative medicine. U.S. studies often focus on rigorous clinical trials and mechanistic investigations, exploring biological and psychological pathways through which Tai Chi alleviates insomnia, thereby responding to the demand for non-pharmacological therapies. The complementary strengths of the two countries—China providing traditional knowledge and clinical data, and the United States contributing methodological rigor and evaluative frameworks—have collectively advanced global progress in this area. Closer collaboration on intervention standardization, mechanistic exploration, and cross-cultural applications could further enhance international impact.Collaboration maps from both databases highlight UCLA and the University of Hong Kong as central hubs in East–West academic partnerships. Professor Michael R. Irwin from UCLA is the most prolific author in this field and maintains the broadest collaborative network.

With regard to journal distribution, the 281 WoSCC articles were published in 178 journals, while the 489 Scopus articles appeared in 283 journals. This wide dispersion highlights the interdisciplinary nature of Tai Chi and insomnia research, spanning medicine, psychology, sports science, and public health. The lack of concentration in a few core journals indicates that the field is still expanding, with findings disseminated across diverse outlets. Nevertheless, Frontiers in Psychiatry and Sleep Medicine Reviews have played central roles in both the volume of publications and citation impact within this field, serving as key platforms for disseminating related research findings. Meanwhile, Sleep has distinguished itself through its high citation frequency and central position within collaborative networks, highlighting its pivotal influence in academic communication and knowledge exchange. The prominent roles of these three journals collectively underscore their indispensable contribution to advancing research on Tai Chi and insomnia.

Research on Tai Chi for insomnia has evolved through three distinct stages, each marked by representative studies. In the early stage (2006–2014), Irwin et al.’s “Cognitive Behavioral Therapy vs. Tai Chi for Late Life Insomnia and Inflammatory Risk: A Randomized Controlled Comparative Efficacy Trial” reflected the exploratory focus of this period, demonstrating the feasibility of Tai Chi and providing a methodological foundation for subsequent research (13). During the middle stage (2015–2019), Du et al.’s “Taichi exercise for self-rated sleep quality in older people: A systematic review and meta-analysis” synthesized multiple RCTs, confirming its efficacy not only in improving sleep but also in enhancing broader health outcomes, thereby consolidating Tai Chi’s position as a core integrative medical intervention (22). In the recent stage (2020–2025), Han et al.’s “Effectiveness of Taijiquan in treating insomnia: A systematic review and meta-analysis of randomized controlled studies” incorporated high-quality evidence and provided more nuanced evaluations, highlighting Tai Chi’s therapeutic value in both sleep and comorbid conditions (11). Together, these studies chart the transition from preliminary testing, to rigorous validation, and finally to advanced evidence synthesis.

### 4.2 Research hotspots and development trends

Using multidimensional bibliometric techniques—including literature clustering, keyword frequency analysis, keyword clustering, Keyword Bursts, and Thematic Evolution—this study identified several major research hotspots in the field of Tai Chi interventions for insomnia. These hotspots can be summarized into three core areas:

#### 4.2.1 Clinical application and efficacy verification of Tai Chi for sleep disorders in specific populations

Tai Chi, a traditional mind-body practice integrating gentle movement, breath regulation, and mindfulness, has increasingly attracted attention for improving sleep disorders across diverse populations. Numerous RCTs and systematic reviews have demonstrated its efficacy and safety in older adults, cancer survivors, patients with chronic pain, and individuals with mood disorders.

Against the backdrop of global aging, insomnia and sleep disorders among the elderly are rising, severely affecting quality of life. Due to its low-impact nature and high adherence, Tai Chi has become a suitable non-pharmacological intervention for older adults. High-quality evidence accumulated over the past two decades strongly supports the significant effects of Tai Chi on improving sleep quality in this population. For example, Siu et al. (2021) reported in an RCT that Tai Chi provided superior long-term benefits for chronic insomnia in older adults compared with conventional exercise (23). Du et al. (2015) found through a systematic review that Tai Chi enhanced sleep quality, life satisfaction, and mental health in older adults (22). Li et al. (2004) conducted a 24-week RCT demonstrating significant improvements in subjective sleep quality, including reduced sleep latency, increased sleep efficiency, and fewer nocturnal awakenings (14). Similarly, Irwin et al. (2008) confirmed that, compared with a health education group, Tai Chi more effectively improved sleep quality and daytime function in older adults with chronic insomnia, with sustained benefits (24). Systematic reviews and meta-analyses by Wu et al. (2015) and Du et al. (2015) synthesized multiple studies, highlighting Tai Chi’s positive impact on overall sleep quality, sleep efficiency, and sleep duration in older adults, as well as its favorable safety profile (23, 25).

Beyond the elderly, Tai Chi has shown significant benefits for cancer patients, who often experience severe sleep disturbances due to disease, treatment side effects, or psychological stress. Irwin et al. (2017) found that Tai Chi improved insomnia in breast cancer survivors comparably to CBT-I, while also reducing fatigue and depressive symptoms (26). Zeng et al. (2014) reported in a systematic review that Tai Chi, as a complementary therapy, significantly improved sleep quality, fatigue, and emotional distress in cancer patients (27). These findings suggest that Tai Chi can directly improve sleep while indirectly promoting recovery by alleviating accompanying symptoms.

In patients with chronic pain, such as those with fibromyalgia or osteoarthritis, Tai Chi’s unique movement patterns and mind-body regulation can effectively reduce pain, thereby improving sleep. For instance, Wang et al. (2010) demonstrated that Tai Chi significantly alleviated pain, fatigue, depression, and sleep disturbances in patients with fibromyalgia, outperforming control interventions (28). Wang et al. (2009) similarly confirmed positive effects on pain and sleep in patients with knee osteoarthritis (29).

Additionally, systematic reviews and meta-analyses by Wang et al. (2010) have shown that Tai Chi serves as an effective adjunct to conventional therapy, aiding patients with mood disorders such as depression and anxiety in restoring sleep continuity (30). Lavretsky et al. (2011) further reported improvements in depressive symptoms and sleep quality among older adults with depression (31).

The application of Tai Chi has also expanded into cardiovascular and neurodegenerative disease populations. For example, interventions improved sleep and overall quality of life in patients with heart failure (32), and preliminary positive results have been observed in individuals with Parkinson’s disease (33).

#### 4.2.2 Mechanistic and evidence-based exploration of Tai Chi for insomnia

Tai Chi, a centuries-old traditional Chinese mind-body practice, has multiple mechanisms through which it improves sleep, and these are increasingly being elucidated by modern science. High-quality evidence continues to support these mechanisms, which is critical for integrating Tai Chi into clinical practice and providing individualized sleep interventions across diverse populations.

Physiologically, the modulation of the autonomic nervous system is a key mechanism underlying Tai Chi’s sleep-promoting effects. Studies indicate that Tai Chi practice can significantly enhance parasympathetic activity while reducing excessive sympathetic arousal, facilitating relaxation crucial for sleep onset. For instance, Lu et al. (2011) demonstrated via heart rate variability (HRV) analysis that even short-term Tai Chi practice immediately increased high-frequency (HF) components in healthy adults, reflecting enhanced parasympathetic activity (34). This autonomic balance aids the transition from daytime stress to nighttime rest, shortening sleep latency and reducing nocturnal awakenings. Tai Chi also modulates the hypothalamic–pituitary–adrenal (HPA) axis, lowering stress hormone cortisol levels (35). Yeh et al. (2017) reported in a systematic review and meta-analysis that Tai Chi improved health indicators in hypertensive patients, indirectly highlighting positive neuroendocrine effects, which are directly beneficial for stress reduction and sleep quality (36). Chronic inflammation and oxidative stress are closely linked to insomnia, and Tai Chi can attenuate systemic inflammation by decreasing pro-inflammatory cytokines (e.g., IL-6, TNF-α) and increasing anti-inflammatory cytokines (e.g., IL-10), while enhancing antioxidant defenses (35). Oh et al. (2020) emphasized in a systematic review and meta-analysis that Tai Chi positively regulates inflammatory biomarkers, providing a physiological foundation for high-quality sleep (37).

Psychologically, Tai Chi incorporates elements of mindfulness meditation. Its mind-body practice requires participants to focus attention on breath and movement, constituting a dynamic mindfulness exercise. This can reduce pre-sleep rumination and anxiety, improving sleep onset. Rusch et al. (2019) confirmed in a systematic review and meta-analysis that mindfulness training effectively enhances sleep quality, aligning well with Tai Chi’s practice characteristics (38). Wang et al. (2014) also reported positive effects of Tai Chi and qigong on depression, with emotional improvement serving as a crucial pathway for alleviating insomnia (30). Tai Chi additionally enhances self-efficacy, empowering patients to actively manage sleep difficulties and reducing helplessness and frustration associated with insomnia.

Social and behavioral mechanisms also contribute to Tai Chi’s effects. As a group practice, Tai Chi provides social support and a sense of belonging, indirectly promoting sleep by alleviating loneliness and improving mental health. Li et al. (2014) highlighted the community-based implementation of Tai Chi interventions in older adults with chronic diseases and emphasized the value of social interaction (39). Furthermore, Tai Chi embodies a healthy lifestyle, encouraging regular routines and moderate physical activity, which helps establish stable circadian rhythms and good sleep hygiene. Irwin et al. (2014) compared Tai Chi with health education and found that Tai Chi offered superior improvements in sleep quality and daytime function for chronic insomnia patients, likely due to its role as a comprehensive lifestyle intervention rather than simple information delivery (35).

In summary, the mechanisms through which Tai Chi alleviates insomnia are multidimensional and interrelated, encompassing physiological regulation of neuroendocrine, autonomic, and inflammatory systems; psychological enhancement of mindfulness, emotion regulation, and self-efficacy; and the promotion of social support and healthy lifestyle behaviors. Future research should deepen the exploration of these mechanisms through high-quality, large-sample, multicenter studies to precisely evaluate dose–response relationships and long-term efficacy in different insomnia types and populations, thereby providing a more robust scientific basis for clinical application.

#### 4.2.3 Comparative and integrative research of Tai Chi and other non-pharmacological interventions

Comparative and integrative studies of Tai Chi and other non-pharmacological therapies help clarify its unique effects and inform the design of personalized, multimodal treatment strategies.

CBT-I is currently considered the gold standard for insomnia treatment (40). Comparative trials indicate that Tai Chi achieves comparable improvements in sleep quality and efficiency, particularly in specific populations such as cancer survivors (41). An early study by Irwin et al. (2014) suggested that Tai Chi was similarly effective to CBT-I in alleviating insomnia in older adults, while potentially offering additional benefits for physical health (13). Importantly, Tai Chi may address physical symptoms that exacerbate sleep problems, such as pain or balance impairments, which are not directly targeted by CBT-I (28, 42).

Compared with traditional aerobic exercise, Tai Chi’s advantage lies in its low-intensity activity combined with high mind-body integration. Research has shown that for individuals with sleep disturbances caused by chronic pain, Tai Chi may outperform conventional aerobic interventions in improving sleep, pain, and functional outcomes (28). A meta-analysis by Song et al. (2003) reported that Tai Chi was more effective than general exercise in enhancing sleep among patients with chronic conditions (43). Tai Chi shares core neurophysiological mechanisms with other mind-body practices, including yoga and qigong, such as breath regulation, interoception, and stress modulation (44). However, the number of head-to-head trials remains limited, and differences in intervention forms and target populations require further investigation.

Integrative strategies represent a rational next step in the field (45). Irwin et al. (2014) proposed a combined CBT-I and Tai Chi intervention aimed at providing comprehensive care for patients with treatment-resistant insomnia (13). Such integrated approaches may be particularly useful for insomnia patients with comorbidities or refractory symptoms, highlighting the potential of Tai Chi within multimodal therapeutic frameworks.

### 4.3 Limitations

The conclusions of this study should be interpreted cautiously within several methodological boundaries, which, while ensuring analytical rigor, also represent inherent limitations.

First, the data sources were limited to the WoSCC and Scopus. This strategy may introduce selection bias, as relevant studies not indexed in these two major databases could have been omitted. Consequently, the findings primarily reflect research trends within the mainstream international academic community.

Second, only English-language publications were included, a decision made for methodological feasibility. Searches of major Chinese databases, including CNKI and Wanfang, revealed a very limited number of studies on Tai Chi interventions for insomnia, insufficient to support robust bibliometric analyses. Conducting such analyses with sparse data can produce unstable or even misleading results. Therefore, to maintain methodological integrity and ensure reliable outcomes, Chinese-language publications were excluded. While necessary, this limitation inevitably introduces geographic and cultural bias into the knowledge representation of the field.

Additionally, due to formatting restrictions in Scopus exports, co-citation network analysis could only be conducted using the WoSCC dataset, which somewhat limits the comprehensiveness of the knowledge base analysis.

Despite these limitations, this study, through clearly defined research boundaries and rigorous data screening, provides a reliable and in-depth quantitative analysis of the international research landscape on Tai Chi interventions for insomnia.

## 5 Conclusion

This study provides a comprehensive analysis of global research trends on Tai Chi interventions for insomnia, revealing sustained growth primarily led by China and the United States. The University of California, Los Angeles (UCLA) and the University of Hong Kong serve as central hubs of collaboration, with Professor Michael R. Irwin from UCLA identified as the most prolific author and maintaining the widest international network in this domain. Frontiers in Psychiatry, Sleep Medicine Reviews, and Sleep have emerged as the leading platforms for dissemination and citation. Research hotspots cluster around three major directions: clinical efficacy in specific populations, mechanistic and evidence-oriented investigations, and comparative or integrative studies with other non-pharmacological interventions. The field has advanced through three stages—early theoretical and methodological exploration (2006–2014), rigorous RCTs (2015–2019), and diversified, precision-oriented development (2020–2025)—reflecting its progression from peripheral interest to mainstream scientific recognition. Collectively, these findings deepen academic understanding, delineate current frontiers, and provide strategic guidance for future innovation in Tai Chi–based insomnia management.

## Data Availability

The original contributions presented in the study are included in the article/**Supplementary Material**. Further inquiries can be directed to the corresponding author.
